# Structured light 3D scanner simulation dataset

**DOI:** 10.1016/j.dib.2026.112566

**Published:** 2026-02-09

**Authors:** Michał Własiuk, Robert Sitnik

**Affiliations:** The Faculty of Mechatronics, Warsaw University of Technology, ul. św. Andrzeja Boboli 8, Warsaw, Poland

**Keywords:** Camera, Projector, Reconstruction, Point cloud, Rendering, Algorithms, 3D analysis

## Abstract

Computer simulation of optical measurement systems plays a crucial role in the design, analysis, and optimization of real-world measurement solutions. However, acquiring real measurement data often involves challenges such as a complex experimental setup, sensitivity to environmental conditions, and the presence of noise and calibration errors, which can hinder controlled algorithm evaluation. To address this issue, in this data article, we introduce datasets generated using a simulated 3D structured light scanner. These samples are produced by projecting a series of sinusoidal and Gray code patterns onto various 3D objects using a simulated projector. The simulated environment also provides the flexibility to apply different surface materials and controlled illumination conditions, enabling systematic testing of algorithms under diverse yet precisely defined scenarios. This approach eliminates real-world uncertainties and errors associated with scene setup and environmental factors, supporting the development and evaluation of point cloud processing and surface reconstruction algorithms in controlled conditions.

Specifications Table

**Table:** 

Subject	Computer Sciences
Specific subject area	Simulated 3D measurement system dataset generated to help in processing algorithm development and evaluation.
Type of data	.png (generated images), .pbrt (pbrt-v4 configuration files), .zip (compressed directories containing .png and .pbrt files)
Data collection	Data generation was performed in a home setup using modified pbrt-v4 software with GPU ray tracing rendering support using Nvidia Optix on 2 PCs with the following specifications: - PC 1: AMD Ryzen 7 9700X, RTX 5070 Ti 16 GB, 64 GB DDR5 6000 MT/s and 4 TB SSD - PC 2: AMD Ryzen 5 5600, RTX 3060 12 GB, 16 GB DDR4 3200 MT/s and 2 TB SSD Datasets were generated using procedural geometry and 3D meshes available in pbrt-v4-scenes. Raw data was stored as generated by the software and then software configuration files were attached for each dataset they correspond.Modified pbrt-v4 and pbrt-v4-scenes repositories are available as archives in the following Zenodo repository:Data identification number: https://doi.org/10.5281/zenodo.18362921Direct URL to data: https://zenodo.org/records/18362921Repositories are preserved in the exact state in which they were used to generate the presented data and contain binary releases for both GPU and non-GPU versions of the pbrt-v4 executable for Ubuntu 24.04 LTS.
Data source location	Faculty of Mechatronics of Warsaw University of Technology, ul. św. Andrzeja Boboli 8, 02–525 Warsaw, Poland
Data accessibility	Repository name: Zenodo Data identification number: https://doi.org/10.5281/zenodo.17826191 Direct URL to data: https://zenodo.org/records/17826191 Data is publicly available in the Zenodo repository without access restrictions.
Related research article	None

## 1 Value of the Data

• The dataset provides data for the development, testing, and evaluation of 3D point cloud processing and surface reconstruction algorithms. It eliminates the need for physical measurement setups by offering simulated image sequences generated under controlled conditions.
• The dataset is generated using procedural geometry and 3D meshes with defined and measurable dimensions. Each scene includes accurate camera and projector calibration data, as well as corresponding ground-truth surface models. These properties allow users to perform quantitative assessments of reconstruction accuracy by directly comparing reconstructed surfaces, depth maps, or point clouds with reference data. The known geometry and precise correspondence between simulated and reconstructed measurements make the dataset suitable for detailed numerical analysis of reconstruction performance.
• The dataset can be used for educational and training purposes in the areas of 3D vision, structured light scanning, and optical metrology. The dataset allows users to experiment with algorithm implementation and visualization of measurement principles without requiring physical equipment. It is suitable for classroom demonstrations, laboratory exercises, and independent study focused on optical measurement systems and 3D data processing.

## 2 Background

The dataset was compiled to support research and development in structured light 3D scanning, a technique that reconstructs object geometry by projecting light patterns – such as Gray codes or sinusoidal fringe patterns – onto surfaces and analysing the resulting deformations captured by cameras. Structured light systems are widely used in applications including industrial inspection [1], cultural heritage preservation [2], medical imaging [3], and human-computer interaction [4]. Generating large-scale, annotated datasets from real-world captures is resource-intensive, requiring specialized hardware, precise calibration, controlled environments, and extensive acquisition time. Additionally, environmental factors such as ambient illumination, surface reflectivity, and occlusions introduce variability that is difficult to reproduce. To address these constraints, the dataset was generated using a simulated structured light acquisition setup. The simulation allows precise control of parameters such as lighting, surface geometry, and camera-projector configurations. The resulting synthetic data provides detailed ground-truth information for each scene, including object geometry and camera-projector calibration, enabling quantitative evaluation of algorithms. The dataset complements research on structured light processing by providing standardized, reproducible data for algorithm development, testing, and training.

## 3 Data Description

The dataset stored in the Zenodo repository [5]. is organized into four compressed subdirectories whose names represent the resolutions of the virtual camera–projector pair used to generate the data these subdirectories store. The naming convention follows *camera-<c_w>-<c_h>-projector-<p_w>-<p_h>* where:

• *c_w* – camera width in pixels,
• *c_h* – camera height in pixels,
• *p_w* – projector width in pixels,
• *p_h* – projector height in pixels.

Additionally, alongside these subdirectories there is also a *README.md* file containing instructions on how to clone and build the code used to generate the data, as well as guidance on how to run the code to recreate the data. Each subdirectory contains three replicas of generated data using three camera models:

• perspective – data acquired using a simulated camera based on the standard perspective model, which is commonly used in visualization applications,
• realistic – data acquired using a simulated camera based on the realistic model described in [6,7],
• realistic with dispersion – data acquired using the same realistic model but extended with camera lens dispersion to evaluate their influence on the final scan.

All camera-type subdirectories share a consistent internal structure that organizes the generated data into clearly defined folders. The _1_CameraCalibrationImages directory contains six images, each depicting a different pose of a fully illuminated calibration board. The _2_PhaseCalculationImages directory follows the same structure as the object scan directories: each scanning pass consists of six sinusoidal images followed by a set of Gray code images, with the number of Gray code images determined by the projector resolution. Each scanning pass is repeated four times at different positions within the measurement volume.

Each object scan directory is named after the scanned object and contains six sinusoidal images followed by a set of Gray code images, again with the number of Gray code images depending on the projector resolution. In addition to calibration and object scan data, each dataset includes a scanner-simulation directory that contains the complete pbrt-v4 configuration files required to reproduce the dataset for the given camera–projector resolution and camera model.

Table 1 summarizes the composition of the datasets for different camera and projector resolution combinations, including camera and projector resolutions, the number of Gray code images, the number of images per object scan, and the total number of images in each dataset aggregated across all scans.

**Table 1:** Dataset composition for different camera and projector resolution combinations.

Dataset name	Camera resolution	Projector resolution	Gray images	Images in scan	Images in dataset
camera–2592–1944-projector-640–480	2592×1944	640×480	8	14	648
camera–4056–3040-projector-1280–720	4056×3040	1280×720	9	15	688
camera–4056–3040-projector-1920–1080	4056×3040	1920×1080	10	16	738
camera–5184–3456-projector-1920–1080	5184×3456	1920×1080	10	16	738

**Fig. 1:**
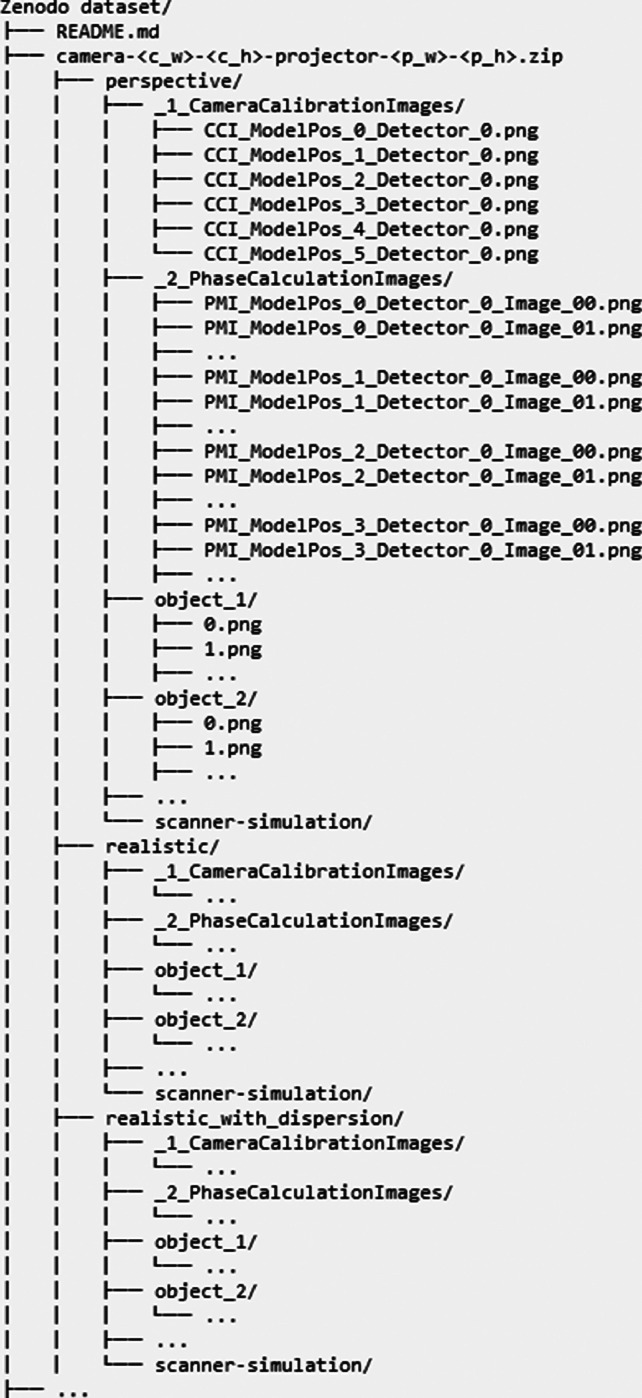
Dataset directory structure tree.

The described directory structure of the presented dataset is shown in Fig. 1. All generated images are stored in .png format to reduce dataset size while preserving full image quality, as PNG is a lossless format that ensures no information is lost during compression. Image naming follows a consistent and structured convention to facilitate automated processing and compatibility with reconstruction pipelines. For object scans, images are indexed sequentially starting from 0, which simplifies processing and usage in custom scripts. In contrast, images in the camera calibration datasets follow a specific naming convention required by the reconstruction software we used to verify that a correct point cloud can be generated from them.

Table 2 shows sample images for each scanned object. The same objects are scanned for each camera type in each camera-projector resolution dataset.

**Table 2:** Sample images of each scanned object for camera-2592–1944-projector-640–480/real-with-dispersion dataset.

Object name	Sample image
_1_CameraCalibrationImages	
_2_PhaseCalculationImages	
crown	
Ganesha	
Gargoyle	
Killeroos	
Killeroos_gold	
LTE-orb	
LTE-orb-white-spec	
MultiSphere	
Planes	
RealFace	
Sphere	

## 4 Experimental Design, Materials and Methods

To accurately simulate the complex interaction between projected light and object surfaces and to produce realistic camera images, we required a suitable rendering framework as the foundation of our method. We selected pbrt-v4 due to its physically based design and high-fidelity simulation capabilities.

To generate datasets that include chromatic aberration caused by wavelength-dependent lens dispersion, we modified the pbrt-v4 codebase. These modifications include the addition of camera lens dispersion support for both CPU and GPU implementations using Sellmeier’s equation, as well as a custom pbrt-v4 configuration file that specifies dispersion constants for each surface of a realistic camera lens. Table 3 illustrates an example of dispersion formula constants for N-BK7 glass – such constants are stored in a configuration file.

**Table 3:** Constants of dispersion formula for N-BK7 glass from SCHOTT glass catalogue.

Constant of dispersion formula	Value
B1	1.03961212
B2	0.231792344
B3	1.01046945
C1	0.006000699
C2	0.020017914
C3	103.560653

For both realistic datasets, we selected a camera model already available in pbrt-v4 that is well documented through a patent application: the d-Gauss F/2, 22° HFOV lens (U.S. Patent 2673,491, Tronnier). Unlike the ideal perspective camera model, which assumes a pinhole projection, the realistic camera model simulates a compound lens system. The realistic model introduces physically based effects such as depth of field due to finite aperture size, optical distortion caused by the bending of light rays as they pass through lens elements away from the optical axis and vignetting caused by partial occlusion of light rays by lens elements at large field angles. For the dataset incorporating dispersion, we used the original patent documentation – which provides refractive indices and Abbe numbers—to construct a file containing the dispersion constants for this lens. These constants were obtained by selecting corresponding glass types from an optical glass catalogue that best match the reported refractive index values.

Scanned virtual objects include geometric models intended to validate various aspects of the reconstruction pipeline. The Sphere, Planes, and MultiSphere are mathematically defined geometries designed to verify geometric accuracy. The Sphere object is intended to evaluate the accuracy of a spherical surface reconstruction. MultiSphere is specifically designed to assess positional error, for example by fitting spheres to the reconstructed data and analyzing the resulting errors in the estimated sphere center locations. Planes evaluate the accuracy and stability of depth reconstruction for planar surfaces.

The remaining objects—RealFace, crown, Ganesha, Gargoyle, Killeroos, Killeroos_gold, LTE-orb, and LTE-orb-white-spec—are scans represented as triangle meshes and are intended to validate how reconstruction software handles complex geometry, fine surface detail, and varying material properties.

The dataset was acquired using 2 PCs equipped with Nvidia GPUs running the GPU version of a modified pbrt-v4 renderer.

## Limitations

The presented dataset is limited to the object geometries, material properties, and lighting configurations defined during simulation; variations outside these parameters are not represented. The dataset is fixed and cannot be modified; however, the underlying simulation software can be configured to generate additional data with alternative patterns, geometries, or acquisition parameters.

## Ethics Statement

The authors have read and followed the ethical requirements for publication in Data in Brief and confirm that the current work does not involve human subjects, animal experiments, or any data collected from social media platforms.

## CRediT Author Statement

**Michał Własiuk:** Software, visualization, methodology, data collection, writing - original draft; **Robert Sitnik:** resources, supervision, writing - review & editing.

## Data Availability

ZenodoSimulated structured light 3D scanner datasets (Original data).
